# The potential anti-tumor effect of anesthetics on cancer by regulating autophagy

**DOI:** 10.3389/fphar.2024.1293980

**Published:** 2024-02-28

**Authors:** Tiantian Wang, Zhixia Zhou, Kai Jiang, Yin Wang, Peifeng Li, Shoushi Wang

**Affiliations:** ^1^ School of Anesthesiology, Weifang Medical University, Weifang, China; ^2^ The Affiliated Hospital of Qingdao University, College of Medicine, Institute for Translational Medicine, Qingdao University, Qingdao, China; ^3^ Department of Anaesthesiology, Qingdao Central Hospital, University of Health and Rehabilitation Sciences (Qingdao Central Medical Group), Qingdao, China

**Keywords:** autophagy, anesthetics, propofol, cancer, opioids

## Abstract

Autophagy is a conserved, cellular self-degradation system that is essential for maintaining intracellular homeostasis. Increasing evidence suggests that autophagy plays an important dual regulatory role in the development of many human diseases, such as cancer. Recent studies have shown that the autophagy process in tumor cells can be regulated by various stimuli from both intracellular and extracellular environments, including the effects of anesthesia. Anesthetics have been shown to not only have clinical anesthetic and sedative effects but also play important roles in the progression of tumors. The effects of different types of anesthetics on tumors differ. In this review, we summarize the basic information on autophagy, the regulatory function of autophagy in cancer, currently used autophagy-targeted tumor therapy, and the effects of different types of anesthetics on tumor progression. We focus on the molecular mechanisms by which anesthetics exert tumor-inhibiting effects by activating or inhibiting autophagy. Herein, we also explore the potential application of the anesthetic/autophagy system in clinical tumor treatment. These findings provide a theoretical basis for the use of anesthetics during the perioperative period to suppress tumor development and provide insights for autophagy-targeted cancer treatment and drug development.

## 1 Introduction

Autophagy is a conserved, cellular self-degradation system that is essential for maintaining intracellular homeostasis. In the 1990s, Professor Yoshinori Ohsumi discovered autophagy-related genes (ATGs) in *Saccharomyces cerevisiae*, marking a milestone in the understanding of autophagy molecules and leading to a better understanding of the autophagy process. Because of these findings, he was awarded the Nobel Prize in Medicine in 2016 for his pioneering work in the discovery of autophagy regulation in yeast. Autophagy was thus proven to be an important metabolic process in eukaryotic cells for the degradation and recovery of damaged macromolecular proteins and organelles (Camuzard et al., 2020). It is also an intracellular process in which cellular contents, such as dysfunctional organelles and macromolecular proteins, are transported to lysosomes for degradation and then recycled by the body, fueling the growth of cells (Kroemer et al., 2010). Therefore, autophagy plays a crucial role in cell quality control and nutrient supply by degrading and repurposing abnormal cellular substances to regulate intracellular homeostasis under physiological and pathological conditions and limits cell damage caused by various diseases (Ohsumi, 1999). An increasing number of studies have shown that autophagy plays an important regulatory role in the occurrence and development of many human diseases, including cancer, neurological diseases, cardiomyopathy, type 2 diabetes, and fatty liver (Lamoureux et al., 2007; Rubinsztein et al., 2012; Nah et al., 2015).

Autophagy exhibits a dual regulatory effect of promoting and inhibiting cancer in the progression of cancer biology. On the one hand, autophagy can limit tumor development by attenuating cellular imbalances and injury in the early stages of cancer. It also exerts an antitumor effect by attenuating chromosomal instability, inhibiting necrosis and inflammation, and promoting cellular senescence (White, 2012). On the other hand, autophagy can also exert protumor effects by restoring intracellular components and increasing energy production to meet the high metabolic requirements of tumor cells in the advanced stages of cancer. Activation of autophagy can lead to an imbalance in the tumor microenvironment and the suppression of immune monitoring, ultimately leading to the growth and metastasis of primary tumors (Viry et al., 2016). Thus, targeted autophagy can regulate the progression, treatment, and even prognosis of various human diseases. Interesting recent studies have shown that autophagy in tumor cells can be influenced by various stimuli both *in vitro* and *in vivo*; these stimuli include cytokines (Ge et al., 2018), microorganisms (Wang et al., 2021), drugs (Klionsky et al., 2021), and even anesthetics (Bundscherer et al., 2015; Yao et al., 2016; Yu et al., 2018; Li et al., 2019; Wei et al., 2021).

An anesthetic is a drug that can make the whole body or localized portion of the body temporarily and reversibly lose sensitivity to pain. Anesthetics are often used for surgery or other medical treatments. The most commonly used narcotic drugs in clinics are intravenous, inhaled, or local anesthetics and opioid analgesics (Smith et al., 2023). Sedation and pain relief are the most basic and important effects of anesthetics. Through clinical experience and the development of new research and technology, new potential clinical applications of anesthetics have been identified. Studies have reported that, in addition to analgesia, anesthetics can inhibit the inflammatory response caused by nociceptive stimuli (Ackerman et al., 2021). Several studies have also shown that the use of anesthetics can improve patient outcomes and inhibit drug resistance. Anesthetics can inhibit the drug resistance of cancer cells by regulating various signaling pathways and expression of various proteins, as well as deoxyribonucleic acid (DNA) methylation and noncoding ribonucleic acid (RNA) expression (Sun et al., 2020; Han et al., 2023). In addition, anesthetics also confer protection to organs (Mei et al., 2022) and show antiepileptic (Migdady et al., 2022) and antipsychotic (Barbic et al., 2021) effects. Notably, in recent years, the antitumor effects of anesthetics have attracted increasing attention. Anesthetics can play an important role in the autophagy, apoptosis, proliferation, and metastasis of some tumor cells (Saito et al., 2019). Through extensive literature research, we have found that autophagy is closely related to tumor progression, and anesthetics can control autophagy by regulating the expression of autophagy related genes to affect tumor progression. Therefore, a better understanding of anesthesia mediated autophagy flow regulation and its impact on tumor occurrence and development will provide new strategies and directions for clinical perioperative anesthesia and treatment of tumors.

This review first presents a summary of the basic information on autophagy (functions, process, and molecular mechanisms) and the regulatory roles of autophagy or anesthetics in cancer. Then, it is focused on the effects of anesthetics on cancer occurrence and development via their regulation of autophagy. In addition, the potential application of anesthetic-mediated autophagy in clinical tumor treatment is discussed. The findings reported to date have enriched the theoretical understanding of anesthetics and autophagy in the development and treatment of cancer and provide a scientific basis for further optimizing and innovating drugs, as well as for rational development of clinical medication, on the basis of key genes and targets.

## 2 Autophagy function, processes, and molecular mechanisms

Autophagy is a major catabolic process in all eukaryotic cells. It regulates the degradation and recycling of long-lived proteins, damaged organelles, and protein aggregates to maintain cell growth and survival (Camuzard et al., 2020). Basal autophagy has been highly conserved throughout evolution, and by eliminating damaged mitochondria, it functions as a “quality control” and cytoprotective mechanism for certain cellular components. Under stress conditions, such as nutrient deprivation, oxidative stress, endoplasmic reticulum stress, or infection, autophagy is stimulated to keep cells alive. Although autophagy is ultimately a survival mechanism, in some cases, it has been shown to cause cell death.

**FIGURE 1:**
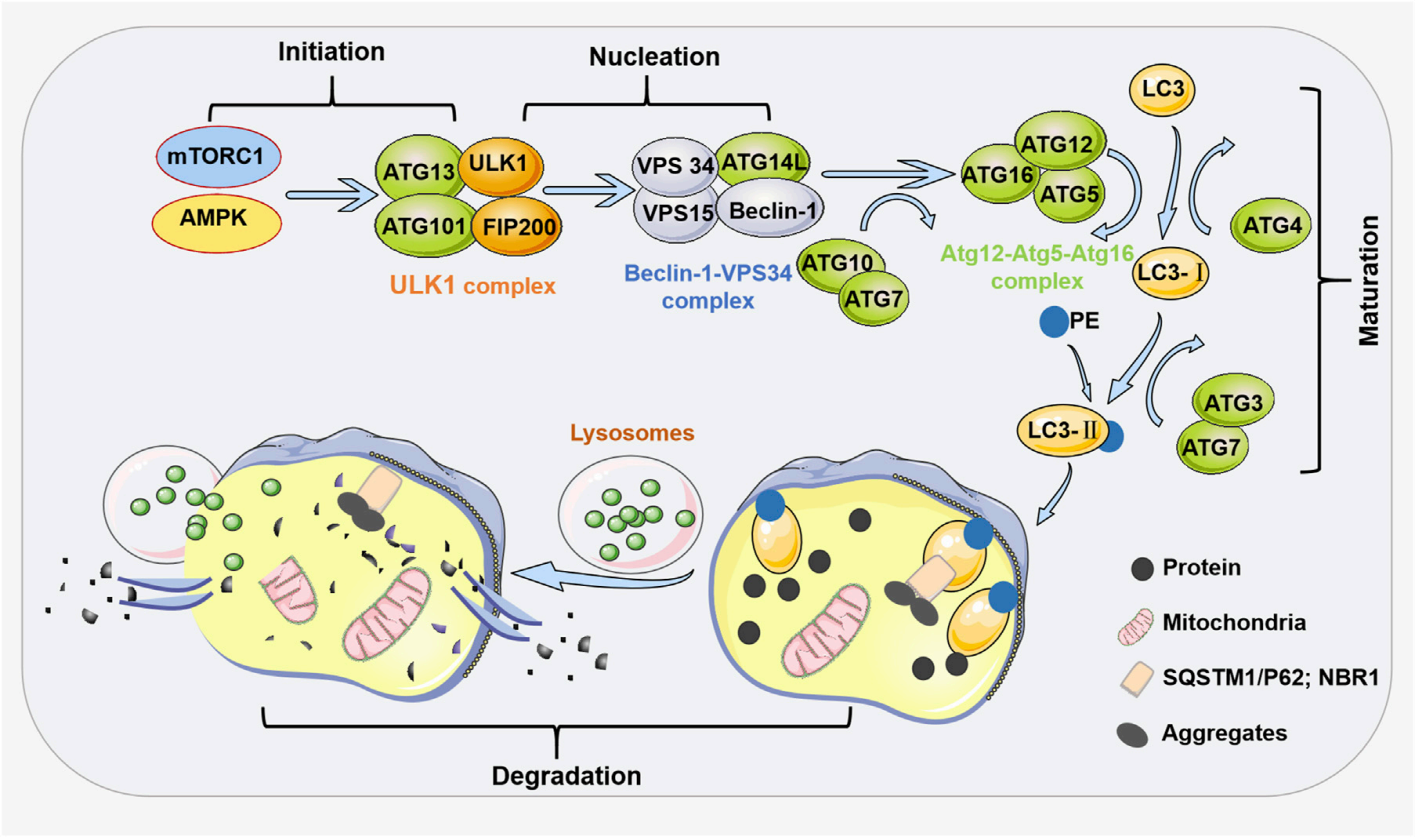
General process steps and regulators of autophagy. At least four steps are involved in the process of autophagy, including initiation, nucleation, maturation, and degradation. The initiation of autophagy is mediated by the ULK1 complex mediated by mTORC1 and AMPK. During the nucleation phase of autophagy, the ULK1 complex phosphorylates and activates the Beclin-1-VPS34 complex. At the maturation stage, the ATG5-ATG12 conjugate and ATG16 form the ATG12-ATG5-ATG16 complex on the autophagosome membrane. This complex recruits the second ubiquitin like system onto the autophagosome membrane. When ATG4 cleaves ubiquitin like protein LC3, LC3 forms a soluble form (LC3-I). Then, ATG7 and ATG3 come into play by adding PE to LC3-I, resulting in LC3-II. In the degradation stage of autophagy, after the formation of autophagosomes, the cargo receptors SQSTM1/p62 and NBR1 recruit the substances required for autophagy degradation to LC3-II. The fusion of autophagosomes and lysosomes forms autophagosomes. During the degradation stage, autophagic substances are degraded by lysosomal hydrolases. The obtained materials are recycled through nutrient carriers to provide fuel for cell growth.

The formation of autophagosomes is a multistep process involving more than 30 ATG proteins, including at least four stages (initiation, nucleation, maturation, and degradation) and many regulators, and involves many regulatory factors and signaling pathways, such as mammalian rapamycin target (mTOR), which is the main signaling pathway (Feng et al., 2014) (Figure 1). In mammals, the initiation of autophagy is mediated by the Unc-51 Like autophagy activation Kinase 1 (ULK) complex (Ganley et al., 2009). The complex consists of ULK1, a FAK-family interacting protein of 200 kDa (FIP200 or ATG17), ATG13, and ATG101 in the cytoplasm (Mercer et al., 2009). This complex is synergistically activated by Sirolimus Complex 1 (mTORC1) and a mechanistic target of AMP-activated protein kinase (AMPK) (Hosokawa et al., 2009). The effect of mTORC1 on autophagy was different under different environmental conditions. Autophagy is inhibited under nutrient-rich conditions because mTORC1 binds to this complex, and mTORC1 phosphorylates and inhibits ULK1 and ATG13 (Ravikumar et al., 2010). In the presence of rapamycin, mTORC1 dissociates from the complex, and under conditions of nutrient deficiency, ULK1 is activated by mTORC1, and ATG13 and FIP200 are phosphorylated, inducing autophagy (Jung et al., 2009). This step, in general, induces the autophosphorylation and activation of ULK1 (Hosokawa et al., 2009). During the nucleation phase of the autophagosome, the ULK1 complex phosphorylates and activates the Beclin-1-VPS34 complex (Kawamata et al., 2008); this complex initiates the formation of autophagosomes, and its components are Beclin-1, VPS34 [a Class III phosphatidylinositol 3-kinase (PI3K)], and other proteins, such as VPS15 and ATG14L (Fimia et al., 2011).

During the maturation stage of autophagosomes, two binding systems (OHSUMI, 1999) and several recruitment proteins are required to establish “ubiquitin-like” complexes (Ohsumi, 1999; Camuzard et al., 2020). The first complex consists of the proteins ATG12-ATG5-ATG16. The second complex includes microtubule-associated protein 1A/1B-light chain 3 (LC3). The ATG12 protein binds to ATG5 through the actions of ATG10 (a ubiquitination E2-like enzyme) and ATG7 (a ubiquitination E1-like enzyme). This ATG12-ATG5 conjugate then forms a complex with ATG16, the ATG12-ATG5-ATG16 complex, on autophagosomal membranes. The ATG12-ATG5-ATG16 complex recruits a second ubiquitin-like complex to the autophagosome membrane. ATG4 cleaves the ubiquitin-like protein LC3 (ATG8), yielding its soluble form (LC3-1) (Kabeya et al., 2000). Then, ATG7 and ATG3 add a phosphatidylethanolamine group (PE) to LC3-I, yielding LC3-II (Kabeya et al., 2004). The ratio of lipidated LC3 (LC3-II) to free LC3 reflects the number of autophagosomes produced over time (Levy et al., 2017). Then, in the degradation phase of autophagy, these ubiquitin-like proteins attach to the autophagosome membrane to recruit cargo receptors that traffic autophagic cargo to the autophagosome. Cargo receptors include SQSTM1/p62 and NBR1. After autophagosome formation, these receptors recruit substances that need to be degraded to LC3-II (Ravikumar et al., 2010). Cargo is enveloped by the isolation membrane, which forms the autophagosome (Ganley et al., 2009). An autophagosome is then transported via microtubules to perinuclear regions with lysosomes (Kawamata et al., 2008). Next, an autophagosome fuses with a lysosome to form an autophagolysosome. Finally, during the degradation phase of autophagy, the autophagic material is degraded by lysosomal hydrolases to produce amino acids, nucleotides, and fatty acids (Tanida, 2011); these substances are recycled through nutrient carriers to provide fuel for cell growth.

The lysosomal degradation pathway in autophagy has evolved to be highly conserved as a nonselective cellular catabolism pathway mediated by ATGs, playing a key role in maintaining cell, tissue and biological homeostasis (Klionsky et al., 2021). Under nutrient deficiency and stress conditions (such as hypoxia, oxidative stress, endoplasmic reticulum deficiency stress, and metabolic stress), autophagy can be activated through pathways related to energy metabolism. Most of these pathways include AMPK and mTORC1 (Ferro et al., 2020). The physiological roles of autophagy include two processes: breakdown of dysfunctional macromolecules to improve cell quality and promote macromolecular recycling to meet the metabolic needs of the organism under nutrient-deficient conditions (Mizushima and Komatsu, 2011; Kim and Lee, 2014). Importantly, there is a clear etiological link between autophagy and human diseases, particularly neurodegeneration, inflammatory diseases and cancers (Levine and Kroemer, 2019).

## 3 Regulatory function of autophagy in cancer

The role of autophagy in cancer is complex and depends on the environmental conditions of the cell (Marsh and Debnath, 2020). Increasing evidence indicates that the tumor microenvironment exerts an important effect on tumor growth, metastasis and progression. Cancer cells may participate in the autophagic circulatory system of intracellular macromolecules to maintain cell growth and metastasis, and acquire resistance to anti-tumor therapy. Here, we summarize the dual role of autophagy in cancer and discuss autophagy-based anticancer therapeutics.

### 3.1 Autophagy plays a double-edged sword role in cancer

**FIGURE 2:**
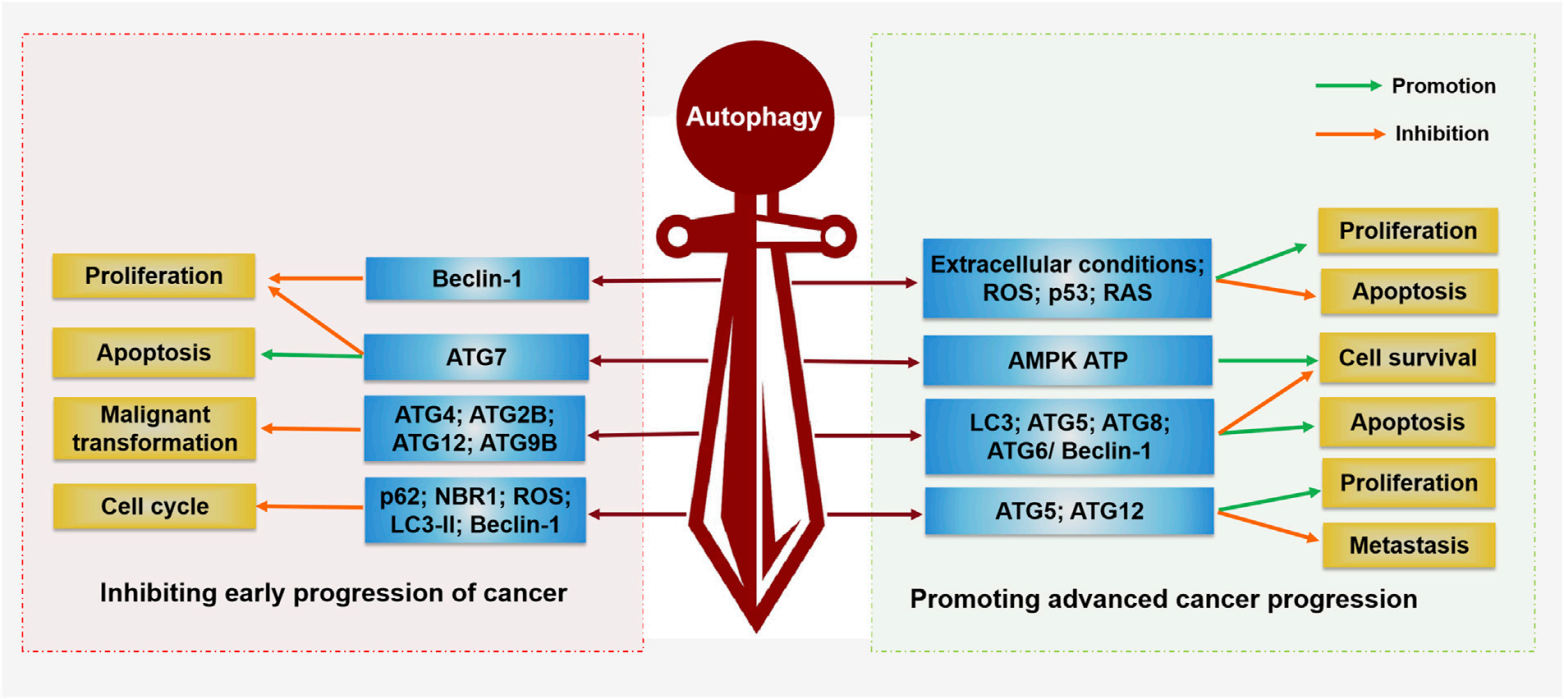
Regulation function of autophagy in cancer. Autophagy plays a double-edged sword in the occurrence and development of cancer. On the one hand, autophagy can inhibit tumor cell proliferation, malignant transformation, and promote cell apoptosis by activating Beclin-1, ATG5, ATG7, ATG4, ATG2B, ATG12, and ATG9B. On the other hand, autophagy can promote the proliferation, survival, and metastasis of tumor cells by activating AMPK, ATP, LC3, ATG5, ATG12, ATG8, ATG6/Beclin-1, ROS, p53, and RAS, while inhibiting tumor cell apoptosis.

Beth Levine’s team first suggested a direct link between autophagy and cancer in 1999 (Kocaturk et al., 2019). They found that deletion of a BECN1/ATG6 single allele in human cells may contribute to malignancy both *in vitro* and *in vivo* (Kocaturk et al., 2019). Moreover, ATGs and related autophagy pathways have been proven to be closely related to oncogenes and tumor suppressor genes (Lamark et al., 2009). In cancer cells, autophagy releases amino acids that are key to cell survival. Under these conditions, autophagy is thought to be a prosurvival mechanism that provides energy to the body by degrading proteins, organelles, and other dysfunctional macromolecules to maintain microenvironment homeostasis (Jose et al., 2018). In addition, autophagy keeps normal cells in balance and reduces normal cell damage to suppress cancer (Zhao et al., 2016). These datas indicate that autophagy has a double-edged sword effect (Figure 2): on the one hand, activation of autophagy can lead to cell death; on the other hand, autophagy can inhibit tumor cell apoptosis and resist the effect of antitumor therapy.

#### 3.1.1 Inhibiting early progression of cancer

Programmed cell death (PCD) is active and orderly cell death that is triggered by gene expression during the development of an organism. When cells are stimulated by internal and external environmental factors, this gene-regulated protective form of cell suicide is initiated, and PCD mechanisms, including autophagy, apoptosis, and necrosis, are the first-line defense to inhibit the proliferation of cancer cells (Ferguson et al., 2015). Therefore, autophagy is involved in cell death mechanisms in addition to that of apoptosis during the early stages of malignant transformation and cancer progression. Deletion of the beclin-1 allele, a key autophagy gene, leads to breast cancer, DNA damage and genomic disorders in mice (Wei et al., 2015). Other autophagy-related proteins have also been found to exert anticancer effects. For example, mice lacking the ATG7 gene showed an increased incidence of tumors (Amaravadi and Debnath, 2014). Loss of ATG5 led to the formation of liver tumors in model mice. Specific partial autophagic protein deletion (Ambra1^+/−^, ATG4c−/−, Sh3glb1^−/−^ and mosaic ATG5^−/−^) in other mouse models was strongly associated with increased tumor incidence (Rybstein et al., 2018). In addition, several studies have observed that mutations in ATG2B, ATG4, ATG5, ATG12, and ATG9B are frequently present in human cancer cells. This evidence suggests that autophagy plays a significant inhibitory role in the malignant transformation of tumors (An et al., 2011; Kim et al., 2011).

#### 3.1.2 Promoting advanced cancer progression

Indeed, during metabolic stress, autophagy can provide nutrients to cancer cells to sustain their survival and development through autophagy and degradation of stromal cells in the tumor microenvironment (Katheder et al., 2017; Yang et al., 2018). Autophagy can regulate interactions between cancer and noncancer cells in the tumor microenvironment (Mowers et al., 2018). In the tumor model, autophagy is activated under certain extracellular conditions (hypoxia, low levels of growth factors and nutrients, and the production of reactive oxygen species (ROS) and lactate) by the oncogenes p53 and rat sarcoma (Ras) to maintain the survival of cancer cells and tumor progression (Poillet-Perez et al., 2015; Brisson et al., 2016). In addition to the activation of autophagy triggered by low levels of nutrients in the tumor microenvironment, ATP generation and AMPK pathways can also tightly regulate autophagy (Ferro et al., 2020). Autophagy is regulated by the presence or absence of metabolic substrates in the microenvironment. Decreased ATP production leads to AMP accumulation, which leads to an increase in AMPK, which in turn activates autophagy. Therefore, autophagy is associated with several signaling pathways involved in tumor development, including fatty acid-, L-glutamine-, and lactic acid-related pathways. For example, in colon cancer cells, autophagy can be activated via the activation of AMPK, which in turn promotes tumor cell proliferation (Wen et al., 2017). Compared with the level in human normal mammary glands, the level of activated LC3 is increased in human mammary ductal carcinoma cells, suggesting that autophagy plays an important role in mammary ductal carcinoma progression (Avivar-Valderas et al., 2011). In addition, evidence from bladder cancer cell studies suggests that ROS may be involved in cancer cell survival and therapy resistance through the activation of autophagy (Amantini et al., 2016). In lung adenocarcinoma cells, low-dose radiation induced the production of ROS, which can activate autophagy and generate radiation resistance (Chen et al., 2015). At different stages of polyoma middle T (PyMT) mammary tumor progression in mice, knockout of ATG5 or ATG12 profoundly inhibited primary tumor growth (Marsh and Debnath, 2020). These datas indicate that inhibiting autophagy by regulating autophagy-regulating genes may become an effective method for treating tumors.

### 3.2 Autophagy-targeting tumor therapy—autophagosome sequestration inhibitors

Autophagy is involved in the metabolic adaptation of cancer cells, and one of the main goals of developing new anticancer therapies is to limit the metabolic adaptation of cancer cells. Therefore, the target-related pathways in autophagy and the related proteins can be used to study the effect of anticancer therapies. To date, an increasing number of researchers have explored autophagy-targeted cancer treatment drugs for specific cancers, and these drugs mainly inhibit autophagy. Two major approaches to autophagy inhibition have been used in mice: 1) knockout of the ATG genes and 2) treatment with lysosomotropic agents that inhibit autophagic flux, such as chloroquine and hydroxychloroquine (Kimmelman and White, 2017). Hydroxychloroquine (HCQ) is the first clinically available autophagy inhibitor and a weak basic lysosomotropic agent. HCQ is a broad-spectrum inhibitor of autophagy associated with chemotherapy or radiotherapy, and its function has been validated in several clinical trials (Ferro et al., 2020). In some patients with solid tumors, HCQ showed the ability to target autophagy safely (Levy et al., 2017). However, its use has been limited by autophagy-independent side effects (Maes et al., 2014). In addition, HCQ activity decreases with acidity. Therefore, we need to explore regulatory mechanisms of autophagy to develop better treatments for cancer. 3-Methyladenine (3-MA), an inhibitor of the autophagy pathway, has been shown to inhibit macroautophagy during the autophagosome sequestration phase in mammalian cells, and it inhibits the activity of PI3K (Petiot et al., 2000). Evidence suggests that 3-MA prevents the formation of pre-autophagosomes, autophagic vacuoles and autophagosomes (Dong et al., 2019). It has been proven that 3-MA can significantly inhibit autophagy induced by tetramethylpyrazine (TET), and 3-MA can inhibit cell growth and apoptosis by inhibiting autophagy (Wang S. et al., 2019). In colon cancer cell experiments, 3-MA synergized with hypoxia to kill colon cancer cells (Li et al., 2010), and the inhibition of autophagy ameliorated this apoptotic effect on HCT116 colon cells. 3-MA attenuated the rapamycin (RAPA; autophagy inducer)-induced death of lung cancer A549 cells by reducing Beclin-1 and LC3-II levels (Yao et al., 2016). When autophagy is induced as a prosurvival mechanism, wortmannin, a specific inhibitor of PI3K, blocks it (Jose et al., 2018). This effect indicates that autophagy inhibitors can suppress the activation of autophagy by acting on autophagy-related factors and thus exert antitumor effects. It has also been suggested that the development of autophagy inhibitors may be effective in the treatment of cancer.

## 4 Regulatory function of anesthetics in cancer

Reducing cancer cell proliferation and inducing their apoptosis are key strategies to prevent tumor growth. Recent studies have shown that anesthetics not only play a role in analgesia and sedation but can also inhibit drug resistance of tumor cells and inhibit tumor progression (Ishikawa et al., 2021; Jing et al., 2022; Saha et al., 2022). To obtain a safe and effective anesthetic intervention, we need to understand the mechanism of these anesthetics to prevent cancer recurrence and promote longer postoperative survival. In the past few decades, numerous *in vitro*, *in vivo*, and clinical studies have demonstrated that anesthetics exert different effects on different cancer types. Anesthetics can modulate the tumor microenvironment by regulating multiple immune mechanisms. Volatile anesthetics, such as sevoflurane, and opioids exert different effects on cancer cells, and these effects are closely related to dose and duration of use (Kim, 2018). However, the vast majority of anesthetics (including different types) have been reported to have potential anti-tumor effects. Moreover, the anti-tumor effects of various anesthetics vary depending on the tumor cell line, tumor location, concentration, and substance properties (Liu et al., 2020; Alam et al., 2021; Jing et al., 2022; Bogár, 2023).

### 4.1 Effects of local anesthetics on tumors

Local anesthetics, such as lidocaine, ropivacaine, levobupivacaine, procaine and bupivacaine, have been proven to reduce tumor growth, proliferation, and metastasis (Kim, 2018). These anesthetics can block voltage-gated sodium channels (VGSCs), which are transmembrane proteins consisting of one pore-forming alpha unit and one or more accessory beta units (Li and Xiong, 2011). As a long-acting local anesthetic, levobupivacaine is widely used as a nerve block, ophthalmic anesthesia, epidural anesthesia and intrathecal anesthesia. The use of levobupivacaine *in vitro* for the treatment of prostate cancer cells has been shown that levobupivacaine exerts potent antiproliferative effects on human prostate cancer cells (Jose et al., 2018). The mode of action of levobupivacaine includes multisite inhibition of adenosine triphosphate (ATP) production. Levobupivacaine is thought to be an activation-suppressive compound in DU145 cells because it inhibits growth of cancer-specific cells but exerts a less profound effect on noncancer cells. However, further analysis is needed based on more cancer cell types and corresponding noncancer cells to fully understand the detailed mechanism of action of levobupivacaine and the specific differences of its actions in cancer cells and noncancer cells. As a local amide-linked anesthetic, bupivacaine is commonly used in cancer patients during tumor resection or in postoperative pain relief and has shown potential anticancer activity against several types of cancer cells (Kim et al., 2015; Kaduri et al., 2021; Mirshahidi et al., 2021; Hao et al., 2022). The mechanism of action of bupivacaine as an anesthetic involves interference of VGSCs (Li and Xiong, 2011; Lucchinetti et al., 2012). It has been reported that bupivacaine can inhibit the growth and proliferation of melanoma cells (Karniel and Beitner, 2000), as well as colon cancer cells (Li et al., 2019), and inhibit the development of gastric cancer via a variety of mechanisms other than sodium channel blockade (Dan et al., 2018). *In vitro*, bupivacaine has shown anticancer activity against pancreatic cancer (Bundscherer et al., 2015). Bupivacaine induces apoptosis in human thyroid cancer cells through the mitogen-activated protein kinase pathway (Chang et al., 2014). In ovarian and prostate cancer, bupivacaine plays an antitumor role by activating apoptotic pathways (Xuan et al., 2016). In addition, lidocaine, another commonly used local anesthetic and antiarrhythmia agent (Svirskis et al., 2020), significantly reduced the invasive ability of osteosarcoma cells and the proliferation of hepatocellular carcinoma cells (Le Gac et al., 2017), while procaine induced apoptosis in cancer cells (Arai et al., 2002). The procaine significantly reduced the invasion and migration of hepatocellular carcinoma cells and inhibited the epithelial-mesenchymal transition (EMT) process by induced c-Met and its downstream oncogenic pathways, such as PI3K/AKT/mTOR and MEK/ERK (Yang et al., 2022). It also reduced tumor burden and abrogated lung metastasis in a mouse model of hepatocellular carcinoma (Yang et al., 2022).

### 4.2 Effect of intravenous anesthetics on tumors

The commonly used intravenous anesthetics in clinical practice include etomidate, ketamine, thiopental sodium, and especially propofol. Propofol (2,6-dipropofol) is a gamma amino butyric acid (GABA) receptor agonist and is a widely used sedative for surgical anesthesia (Sahinovic et al., 2018). It has been reported to play an important role in the tumor cell cycle, proliferation, apoptosis, EMT, migration, metastasis, and angiogenesis (Gao et al., 2020). Propofol reduced the production of interleukin-6 (IL-6) and IL-8 in trastuzumab-resistant breast cancer cells while inhibiting the formation of mammospheres and the EMT (Tian et al., 2020). It has been found to increase the level of the tumor suppressor miR-149-5p and downregulate IL-6 expression, thus making trastuzumab-resistant breast cancer cells sensitive to epigenetic modifications (Tian et al., 2020). Thus, propofol can improve the sensitivity of cells to a variety of chemotherapeutic agents and may be effective in cancer patients receiving chemotherapy interventions.

### 4.3 Effects of volatile anesthetics on tumors

Volatile anesthetics affect tumor progression by inducing apoptosis and inhibiting the proliferation of immune cells such as T lymphocytes and natural killer (NK) cells (Matsuoka et al., 2001). Isoflurane, which is commonly used in the clinic, has a strong anesthetic effect, is quick acting and is rapidly eliminated. It regulates the tumor microenvironment by controlling tumor cell proliferation, immune and inflammatory responses, the EMT, cell migration and metastasis, and angiogenesis. Isoflurane can alter the mitochondrial membrane potential of glioma cells, resulting in increased the activity of electron transport chain complexes I, II, and III and decreased the activity of complex IV to induce mitochondrial damage and apoptosis (Lin et al., 2020). Studies have found that isoflurane suppresses proliferation, migration, and invasion and facilitates apoptosis in colorectal cancer cells through targeting miR-216 (Cai et al., 2021).

### 4.4 Effects of opioid analgesics on tumors

Opioid analgesics are commonly used in the clinic may regulate tumor development by regulating cell proliferation and death. During the course of opiate alkaloid treatment, many immunocompetent cells express opioid receptors, inhibit the immune response and induce apoptosis. However, opioids play different roles in different cancer cell types. In breast cancer cells expressing wild-type p53, morphine increased the production of p53-dependent proteins, including p21, recombination Bcl-2 associated X protein (Bax) and TNF receptor superfamily, member 6 (FAS), by inducing p53 phosphorylation (Kim, 2018). These outcomes suggest that morphine may inhibit the progression of some cancers by activating p53. Morphine administration before and after surgery decreased the tumor-promoting effect of surgery in rats (Page et al., 1993). In addition, morphine treatment before and after operation significantly reduced the elevated level of operation-induced corticosterone in rats (Page et al., 1998). This finding suggests that preoperative morphine analgesia may play a key inhibitory role in surgery-induced metastasis. However, morphine has also been reported to have a tumor-promoting effect, which may be related to different tumor types and stages (Brinkman et al., 2018; Bimonte et al., 2019; Wang et al., 2022).

## 5 The antitumor effects of anesthetics are mediated by autophagy regulation

**FIGURE 3:**
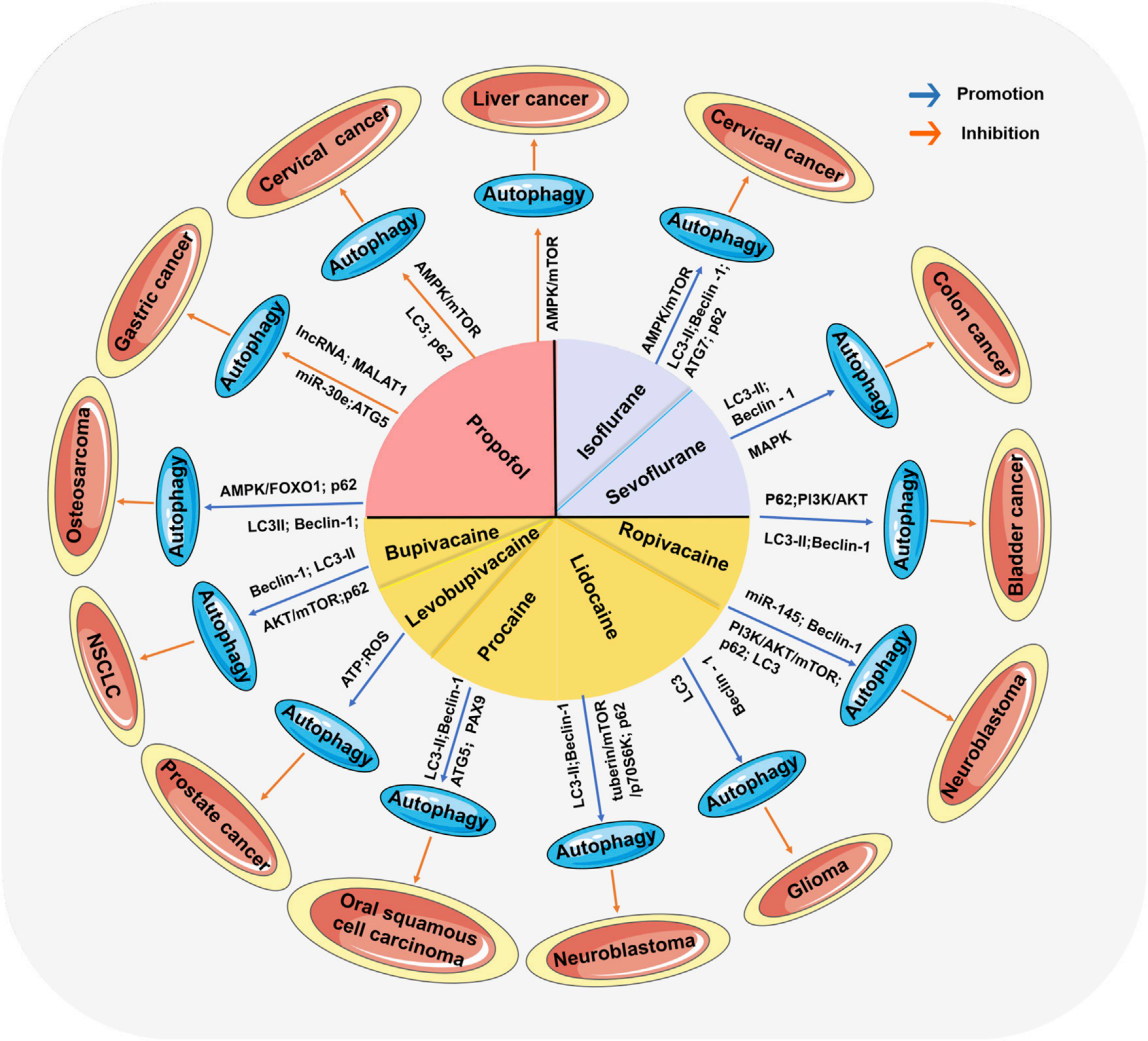
The comprehensive mechanism of anesthetics affecting tumors by regulating autophagy. Anesthetics mediate the activation or inhibition of autophagy through many signaling pathways, thereby affecting the progression of various tumors.

The high morbidity and mortality of malignant tumors is an important problem threatening human life and health. To date, radical surgery, radiotherapy, chemotherapy and hormone therapy are used for treatment, but it is still difficult to prevent the recurrence and metastasis of cancer (Liu et al., 2022a; Liu et al., 2022b; Zhou et al., 2022). The clinical prognosis of patients with malignant tumor is directly influenced by whether the tumor metastasizes, whether the tumor recurs and the mortality. The clinical prognosis depends on the balance between the ability of anti-tumor and the ability of proliferation and invasion of tumor cells. A growing body of experimental data suggests that anesthetics also have the ability to directly influence cancer cell phenotype and metastatic potential, and thus may disrupt this balance at critical moments of immune susceptibility, thus affecting clinical prognosis (Perry et al., 2019). Thus, anesthetics are potential prognostic factors of malignant tumors and it reveals the complex and variable roles of anesthetics and autophagy in cancer progression and treatment (Tan et al., 2015). Interestingly, most anesthetics that affect autophagy have been reported to exert antitumor effects on tumors. More importantly, the antitumor mechanisms of action of different types of anesthetics have different effects on autophagy regulatory programs by either inducing their inhibition or activation (as shown in Table 1; Figure 3). Many studies have shown that many signaling pathways are involved in the regulation of autophagy (Wang H. et al., 2019; Zhang et al., 2019; Yang et al., 2020). Among these pathways, the main focus is on mTOR, a protein kinase that regulates cell growth, survival, immunity, and metabolism. The activation of the mTOR pathway can promote tumor growth, proliferation, and metastasis (Hua et al., 2019). mTOR plays a crucial role in regulating the activation of autophagy (Kim and Guan, 2015). Anesthetics have been reported to affect autophagy and subsequently tumor progression by mediating mTOR signaling. Moreover, even the same general anesthetic, such as propofol, can promote or inhibit autophagy under different conditions (Zhang et al., 2020; Dai et al., 2022).

**TABLE 1:** The regulatory effect of anesthetics on cancer by promoting or inhibiting autophagy.

Anesthetics	Administration methods	Roles in cancer	Regulations in autophagy	Related cellular process	Related genes and pathways	Tumor types	Refs
Propofol	5/10/20 μM, 24 h, *in vitro*; 35 mg/kg, 30 days, *in vivo*, *i*.*p*	Inhibition	Anti-autophagy	Proliferation; Cell cycle; Apoptosis	AMPK/mTOR	Liver cancer	Wang Y. et al. (2020)
	400/800 μM, 3/24 h, *in vitro*	Inhibition	Anti-autophagy	Apoptosis	AMPK/mTOR; LC3; p62	Cervical cancer	Chen et al. (2018)
	4 μg/mL, 2 h, *in vitro*; 4 μg/mL, 4 w, *in vivo*, *i*.*p*	Inhibition	Anti-autophagy	Apoptosis; Chemotherapy resistance	MALAT1; miR-30e; ATG5	Gastric cancer	Zhang et al. (2020)
	0/2.5/5/10 μg/mL, 24 h/48 h/2 w, *in vitro*	Inhibition	Pro-autophagy	Proliferation; EMT; Invasion; Metastasis	AMPK/FΟΧO1; LC3II; Beclin-1; p62	Osteosarcoma	Dai et al. (2022)
Lidocaine	0/0.1/0.5/1/2/4/8 mM, 4/24/48 h, *in vitro*	Inhibition	Pro-autophagy	Apoptosis; Proliferation	PI3K/AKT/mTOR; miR-145; Beclin-1; p62; LC3	Neuroblastoma	Wang Z. et al. (2020)
Bupivacaine	1mm, 0/12/24/48/72 h, *in vitro*; 40 mmol/kg, 28 days, *in vivo*, *s*.*c*	Inhibition	Pro-autophagy	Apoptosis; Proliferation; Invasion; Metastasis	AKT/mTOR; Beclin-1; LC3; p62	Non-small-cell lung cancer	Gu et al. (2021)
Levobupivacaine	1mM, 0/24/48/72 h, *in vitro*	Inhibition	Pro-autophagy	Cell cycle	ATP; ROS	Prostate cancer	Jose et al. (2018)
Ropivacaine	0/2.5/5/10/20/40/80 μM, 24 h, *in vitro*	Inhibition	Pro-autophagy	Apoptosis; Invasion; Proliferation; Metastasis	PI3K/AKT; LC3; Beclin-1; p62	Bladder cancer	Qiao et al. (2023)
Lidocaine	0.25/0.5/1/5/10/15/30 mM, 24 h, *in vitro*	Inhibition	Pro-autophagy	Apoptosis	Beclin-1; LC3	Glioma	Izdebska et al. (2019)
Sevoflurane	1.7/3.4/5.1%, 6/24/48 h, *in vitro*; 1.7/3.4/5.1%, 21 d, s.c.	Inhibition	Pro-autophagy	Cell cycle; Proliferation; Invasion; Apoptosis; EMT	LC3; Beclin-1; MAPK	Colon cancer	Zhang and Shao (2016)
Procaine	0.1/0.5/1 mM, 72h, *in vitro*	Inhibition	Pro-autophagy	Proliferation; Apoptosis	LC3; Beclin-1; ATG5; PAX9	Oral squamous cell carcinoma	Bhol et al. (2022)
Procaine	0/2.1/2.5/2.8/3.2/3.5 mM, 24 h, *in vitro*	Inhibition	Pro-autophagy	Proliferation; Morphological abnormalities in cells	Beclin-1; LC3; p62; tuberin/mTOR/p70S6K	Human neuroblastoma SH-SY5Y	Xiong et al. (2017)

*i.p*: intraperitoneal injection; *s.c*: subcutaneous injection.

### 5.1 Inhibiting autophagy mediated by intravenous anesthetics

Recent studies have confirmed that autophagy inhibition plays an important role in the antitumor effect of intravenous anesthetics, especially propofol (Wang Y. et al., 2020; Li et al., 2020; Zhang et al., 2020; Liu et al., 2023). Propofol can reduce the proliferation of cancer cells and induce their apoptosis by regulating growth family member 3, caspase, glycogen synthase kinase 3 β, and the mitogen-activated protein kinase pathway in breast cancer, lung cancer, pancreatic cancer, and skin cancer (Du et al., 2013; Chen et al., 2017). The molecular mechanisms underlying propofol-induced cell apoptosis mainly include reducing the expression level of miRNAs, inhibiting the signal transduction mediated by long-chain noncoding RNAs (lncRNA), and blocking the production of ROS (Shang et al., 2018; Yu et al., 2018; Wang H. et al., 2020; Ren and Zhang, 2020).

The regulation of autophagy by propofol is often achieved by its regulatory effect on autophagy-related genes or pathways. AMPK is a widely expressed key regulator of autophagy that not only can directly promote autophagy by phosphorylating autophagy-related proteins such as mTOR and Unc-51 but can also indirectly promote autophagy by regulating the expression of autophagy-related genes downstream of transcription factors (Li and Chen, 2019). In tumors, AMPK has also been reported to be a novel target for the resistance of cancer to chemotherapy (Bort et al., 2019). The inhibition of glycolysis mediated by enhanced AMPK/mTOR signaling pathway signaling inhibits the activity of glycogen synthase kinase-3 beta (GSK-3β), which can suppress the acquisition of the malignant hepatocellular carcinoma (HCC) phenotype (Fang et al., 2019). The intravenous anesthetic propofol activated AMPK to inhibit the growth of human hepatocellular carcinoma (HEPG2) cells *in vitro* and prevented the occurrence of transplanted liver cancer in mice by inducing autophagy (Wang Y. et al., 2020). In HeLa human cervical cancer cells, propofol exerted its antitumor effect by inducing endoplasmic reticulum (ER) stress and regulating the AMPK/mTOR signaling pathway to block autophagosome–lysosome fusion and promote autophagosome accumulation (Chen et al., 2018). ER stress refers to the destruction of the oxidative environment in the endoplasmic reticulum lumen and the disorder of ER function when cells are affected by some factors, such as hypoxia and drug toxicity. It causes the accumulation of unfolded or misfolded proteins in the endoplasmic reticulum lumen and the imbalance of calcium balance (Almanza et al., 2019).

In addition to AMPK, LC3 and ATG5 have been confirmed to be involved in the propofol-induced regulation of autophagy in tumor cells. In the gastric cancer context, the intravenous anesthetic propofol was shown to downregulate lncRNA metastasis-associated lung adenocarcinoma transcript- 1 (MALAT-1) and inhibit ATG5 to suppress autophagy and induce apoptosis, thereby enhancing the chemosensitivity of chemotherapy-resistant gastric cancer cells to cisplatin (Zhang et al., 2020). Propofol inhibited the resistance of GC cells to chemotherapy by inhibiting the expression of LC3 and ATG5 (Zhang et al., 2020). In HeLa cells, the level of p62 was increased, and the number of GFP-LC3 fluorescence dots was significantly increased after propofol stimulation. Moreover, the unfolded protein response (UPR) was activated in HeLa cells, which induced the accumulation of autophagosomes in HeLa cells to promote cell death (Chen et al., 2018).

However, some experiments have shown that propofol inhibits tumor progression by promoting autophagy. For example, in a human osteosarcoma experiment, propofol was reported to promote autophagy by upregulating LC3II/I and Beclin-1 protein expression and downregulating the expression of p62 protein via AMPK/FOXO1 signaling pathway, thus inhibiting the proliferation, migration and invasion of tumor cells (Dai et al., 2022).

### 5.2 Promoting autophagy mediated by local and inhaled anesthetics

The antitumor effect mediated by local and inhaled anesthetics is achieved mainly by autophagy promotion. Among these mechanisms, the activation of autophagy via the antitumor effect of local anesthetics is usually related to the AKT/mTOR signaling pathway (Hua et al., 2019). AKT/mTOR signaling, a key axis in cancer development and a potential drug therapeutic target, can induce cell proliferation at different stages of cell development (Zhu et al., 2015). For example, in NSCLC cells, significantly increased rates of AKT/mTOR signaling pathway activation led to the inhibition of autophagy in cancer cells and the promotion of tumor progression (Wang et al., 2016), suggesting the AKT/mTOR signaling axis as a potential therapeutic target for tumors. Lidocaine, which reduces tumor cell viability in a time- and dose-dependent manner, has been reported to inactivate the PI3K/AKT/mTOR signaling pathway and promote autophagy (Hua et al., 2019). Lidocaine has also been proven to induce upregulated miR-145 expression (Wang Z. et al., 2020). Further study revealed that miR-145 targeted AKT3 to inhibit PI3K/AKT/mTOR pathway activation (Wang Z. et al., 2020). In a word, this report suggests that lidocaine promotes autophagy in neural cells to inhibit tumor development by regulating the expression of miR-145 and further inactivating the PI3K/AKT/mTOR signaling pathway.

Bupivacaine inhibited tumor cell proliferation and migration through AKT/mTOR signaling pathway to increase the expression of Beclin-1 and the expression ratio of light chain 3B-II (LC3B-II)/LC3B-I in NSCLC cells (Gu et al., 2021). In addition, bupivacaine decreased the expression of the autophagy-conjugating protein p62 (Gu et al., 2021). During autophagosome formation, P62 acts as a bridge between LC3 and proteins that are selectively encapsulated in the autophagosome and subsequently degraded by proteolytic enzymes in the autophagolysosome. Therefore, the expression of p62 protein is negatively correlated with autophagy activity, which suggests that bupivacaine could induce the activation of autophagy. Corresponding research has confirmed that the autophagy inhibitor 3-MA reversed these changes induced by bupivacaine in NSCLC cells (Gu et al., 2021). In human prostate cancer (DU145) cells, levobupivacaine was involved in the inhibition of glycolysis and oxidative phosphorylation, leading to a decrease in cellular ATP and the production of ROS, thereby inducing autophagy activation to mediate the anticancer effects of this local anesthetic, while it had no effect on noncancerous cells (Jose et al., 2018). When combined with wortmannin, a specific inhibitor of PI3K and an inhibitor of autophagy, the antitumor effect of bupivacaine was profoundly weakened (Jose et al., 2018). These data deepen the understanding of the relationship between autophagy activation and levobupivacaine and indicate the potential value of levobupivacaine as an inhibitory compound for sensitive prostate cancer cells.

Similarly, in ropivacaine-mediated antitumor experiments, Beclin-1 levels and the LC3-II/LC3-I ratio were increased, while p62 expression was decreased in bladder cancer cells after treatment with ropivacaine (Qiao et al., 2023). Ropivacaine has also been shown to inhibit the proliferation and migration of bladder cancer cells by inhibiting the PI3K/AKT signaling pathway to promote autophagy (Qiao et al., 2023). In rat C6 glioma cells, lidocaine protects the organism from cancer by increasing the transcriptional level of LC3B and Beclin-1 and promoting the formation of cytoprotective autophagy (Izdebska et al., 2019).

In oral squamous cell carcinoma, procaine could inhibit DNA methyltransferase and increases PAX9 expression in cells (Bhol et al., 2022). Treatment with procaine led to increased transformation of LC3-II and increased expression of autophagy proteins Beclin-1 and ATG5, which in turn triggered apoptosis and autophagy and inhibited the proliferation and differentiation of cancer cells (Bhol et al., 2022). In addition, in human neuroblastoma SH-SY5Y cells, local anesthetics including procaine inhibited tuberin/mTOR/p70S6K signaling pathway, a negative regulator of autophagy activation, leading to an increase in LC3-II levels, a decrease in P62 levels, upregulation of autophagy flow, and an increase in autolysosomes, thereby triggering autophagy activation, which may be protective mechanisms against local anesthetic neurotoxicity (Xiong et al., 2017).

Similar to local anesthetics, the antitumor effect mediated by inhaled anesthetics is also mainly achieved by promoting autophagy. Isoflurane not only upregulated LC3-II/I, Beclin-1, and ATG7 and downregulated p62 but also induced oxidative stress and activation of the AMPK/mTOR pathway to promote apoptosis and autophagy and inhibit the proliferation of cervical cancer cells (Wei et al., 2021). Isoflurane also upregulated the expression of mammalian target proteins in the protein kinase B/mammalian target of rapamycin signaling pathway and induced NSCLC migration (Zhang and Shao, 2016). In a colon cancer experiment, an increase in sevoflurane concentration resulted in a decrease in the LC3 I/LC3 II ratio in colon cancer cell lines (Yang et al., 2019). In addition, the LC3 protein level was found to be increased in colon cancer cells, suggesting an increase in autophagy (Yang et al., 2019).

## 6 The anesthetic/autophagy system may serve as a potential cancer treatment strategy

Autophagy has been proven to intersect with almost all cancer-related signaling pathways at multiple levels. For example, mTOR, MYC, and Ras are among the most important signaling pathways that are frequently exploited by cancer cells to reprogram the body’s metabolism, cell survival, protein and organelle switching, and bioenergy functions (Qiu and Simon, 2015). These observations suggest that autophagy plays a complex and dynamic role in cancer, which may explain the dual nature of autophagy during cancer progression (Kimmelman and White, 2017). Indeed, increasing evidence shows that autophagy inhibits tumor progression in the early stages of malignant transformation and cancer progression, whereas in the later stages, autophagy exhibits a catalytic effect to promote tumor growth and resistance to treatment. To date, many types of autophagy sequestration inhibitors have been developed, studied, and gradually applied in clinical practice to mitigate the excessive activation of autophagy in tumor cells. In general, many tumors are in middle and late stages progression when they are discovered. However, with the advancement of technology and the discovery of new tumor-specific detection molecules, the early detection and treatment of tumors are becoming increasingly attainable objectives. Therefore, the activation and regulation of autophagy in the early stages of tumors, as well as the development and application of targeted drugs, urgently need to be given attention by researchers.

There is still significant controversy over the relationship between anesthetic drugs and malignant tumors, as anesthetic drugs have been proven to have both antitumor effects and tumor development-promoting effects, which to a certain extent depend on the type of drug, anesthesia method, and tumor type. However, it is interesting to note that, as mentioned above, anesthetics that regulate autophagy in tumor cells are mainly antitumor agents. Therefore, based on the characteristics of autophagy regulation mediated by anesthetics, we propose the following considerations for an anesthetic/autophagy system for cancer therapy. In early tumor resection surgery, specific anesthetics that activate autophagy, such as local and inhaled anesthetics, can be used to block the synthesis of cancer-related proteins and inhibit tumor progression. However, in the treatment of advanced tumors in patients with cancer who are receiving sedatives and cancer pain medication and, especially, those undergoing cancer resection surgery, intravenous anesthetics such as propofol, which mainly inhibit autophagy, are preferred to help control postoperative recurrence and metastasis. Recent clinical studies have shown that cancer patients who receive propofol as a total intravenous anesthesia (propofol-TIVA) have a better prognosis and a longer survival period than those who receive anesthesia via inhalation (Li et al., 2018; Wang et al., 2018; Yap et al., 2019; Chang et al., 2021). Although research on anesthesia/autophagy system therapy is still in the exploratory stage, with increasing understanding of cancer cell autophagy mechanisms and the development of new anesthetics, more effective treatment methods may emerge in the future.

## 7 Conclusion and discussion

In this review, we conclude that anesthetics can induce changes in autophagy factors that regulate tumor-related gene expression, thereby affecting tumor progression. Under physiological conditions, autophagy plays a role in degrading cellular substances and providing energy to maintain body stability. Under the conditions of some diseases, autophagy is influenced by signaling pathways and the cell cycle, which eventually begin to function abnormally. In the future, we need to further study the translation and posttranslational modification levels of autophagy genes. Autophagy exerts a profound influence on the development of many diseases, including cancer. Cancer has heterogeneous causes, and autophagy is a double-edge sword in cancer cells. Furthermore, there is increasing evidence that some autophagy-related molecules, such as ATG proteins and LC-3, are associated with pathological variables or clinical outcomes of patients with diseases, and therefore, as autophagy biomarkers, they may have important clinical value. In addition, multiple studies have demonstrated that anesthetics can regulate cancer initiation and progression by affecting autophagy-related factors such as the ATG family, LC-3, and others. These findings suggest the prospect of anesthetics in the treatment of cancer. Indeed, the precise molecular mechanisms by which autophagy affects human health require further investigation. The application of anesthetics/autophagy systems in clinical medicine faces many challenges. First, whether autophagy is truly involved in the development of these diseases or is a byproduct of other molecular mechanisms needs to be clarified. It needs to be demonstrated that the progression or inhibitory effect of anesthetics on cancer cells may be due to the activation or inhibition of the immune system. Of course, the impact of anesthesia on cancer may also be a positive side effect that occurs during surgery, so using anesthesia alone as a special cancer treatment drug may not be feasible. Therefore, more research is needed to rule out the impact of various factors such as immune system activation and surgical emergency on the anti-tumor effect of anesthetics. Second, we need to better understand the mechanisms of autophagy to a large extent, and some study results have suggested relatively clear molecular mechanisms. Third, in clinical practice, the potential applications of autophagy and its inhibitors as diagnostic and therapeutic targets are still in the theoretical and experimental stages, and ways to determine the most efficacious drug administration modality, side effects, drug resistance, and treatment efficacy are needed. Moreover, it must be said that there are often conflicting views in current reports on the complex mechanisms of autophagy, cancer, and anesthetics. This makes it difficult for us to draw universal conclusions that contribute to the relationship between them, and also makes it difficult for us to reconcile these data in order to draw more reasonable pictures. In addition, we need to explore the appropriate clinical drug concentrations used in cancer surgery to unleash the potential positive effects of anesthetics. Therefore, we should conduct further research and clinical trials to fully understand the regulatory mechanisms and potential clinical applications of anesthetic/autophagy systems.

## Figures and Tables

**Table:** 

**ATGs**	autophagy-related genes
**AKT**	protein kinase B
**AMPK**	AMP-activated protein Kinase
**ATP**	adenosine triphosphate
**Bax**	recombinant Bcl-2 associated X protein
**DNA**	deoxyribonucleic acid
**ER**	Endoplasmic reticulum
**EMT**	epithelial-mesenchymal transition
**FAS**	TNF receptor superfamily, member 6
**FIP200**	FAK-family interacting protein of 200 kDa
**GABA**	gamma amino butyric acid
**GSK-3β**	glycogen synthase kinase-3 beta
**HCC**	hepatocellular carcinoma
**HCQ**	Hydroxychloroquine
**HEPG2**	human hepatocellular carcinomas
**IL-6**	Interleukin-6
**IL-8**	Interleukin-8
**LC3**	1A/1B-light chain 3
**LC3B-II**	light chain 3B-II
**LncRNA**	long non-coding RNA
**MALAT-1**	metastasis-associated lung adenocarcinoma transcript 1
**mTOR**	mammalian target of rapamycin
**mTORC1**	Sirolimus Complex 1
**NK**	natural killer
**NSCLC**	non-small-cell lung carcinoma cells
**PCD**	programmed cell death
**PI3K**	phosphatidylinositol 3-kinase
**PyMT**	polyoma middle T
**RAPA**	rapamycin
**Ras**	rat sarcoma
**RNA**	ribonucleic acid
**ROS**	reactive oxygen species
**TET**	tetramethylpyrazine
**ULK1**	Unc-51 Like autophagy activation Kinase 1
**UPR**	unfolded protein response
**VGSC**	voltage-gated sodium channels
**3-MA**	3-methyladenine
